# Supplementary material for: MSFragger-DDA+ enhances peptide identification sensitivity with full isolation window search
Source: Nat Commun. 2025 Apr 8;16:3329. doi: 10.1038/s41467-025-58728-z (PMC11978857; doi:10.1038/s41467-025-58728-z)
Supplement: Supplementary file 1 — Supplementary Information [file 41467_2025_58728_MOESM1_ESM.pdf]

Supplement to: “MSFragger-DDA+ Enhances Peptide Identification Sensitivity with Full Isolation Window Search"

Fengchao Yu[1*], Yamei Deng[1], and Alexey I. Nesvizhskii[1,2*]

1. Department of Pathology, University of Michigan, Ann Arbor, MI, USA.

2. Gilbert S. Omenn Department of Computational Medicine and Bioinformatics, University of Michigan, Ann Arbor, MI, USA.

*Corresponding Authors: yufe@umich.edu, nesvi@umich.edu

Tool	Target sequences	Entrapment sequences	FDP estimation upper bound	FDP estimation lower bound
MaxQuant	31146	54	0.35%	0.17%
MetaMorpheus	44319	689	3.06%	1.53%
MSFragger-DDA FragPipe	42726	22	0.10%	0.05%
MSFragger-DDA+ FragPipe	68629	45	0.13%	0.07%

Supplementary Figure 1. Peptide-level FDP evaluation for MaxQuant, MetaMorpheus, MSFragger, and MSFragger-DDA+. Two calculation methods, including the upper bound and lower bound method, were applied. Source data are provided as a **Source Data** file.

a

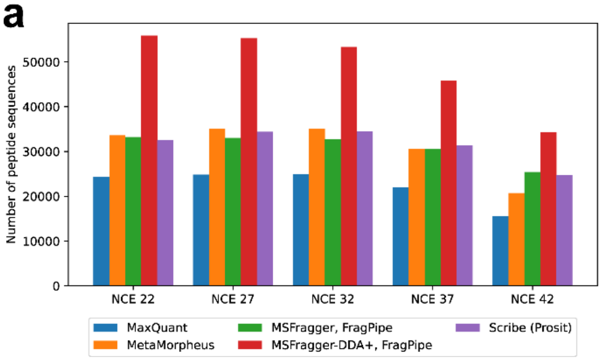

b

Tool	Target proteins	Entrapment proteins	FDP estimation	
			upper bound	lower bound
MaxQuant	3509	27	1.53%	0.76%
MetaMorpheus	4348	66	2.99%	1.50%
MSFragger FragPipe	4522	16	0.71%	0.35%
MSFragger-DDA+ FragPipe	5656	29	1.02%	0.51%

Supplementary Figure 2. Sensitivity and false discovery proportion evaluation similar to Figure 2, but the latest version of MetaMorpheus (version 1.0.6) is used. (a) Number of peptide sequences identified by MaxQuant, MetaMorpheus (version 1.0.6), MSFragger, MSFragger-DDA+, and Scribe. **(b)** Protein-level FDP evaluation for MaxQuant, MetaMorpheus (version 1.0.6), MSFragger, and MSFragger-DDA+. Two calculation methods, including the upper bound and lower bound, were applied. Source data are provided as a **Source Data** file.

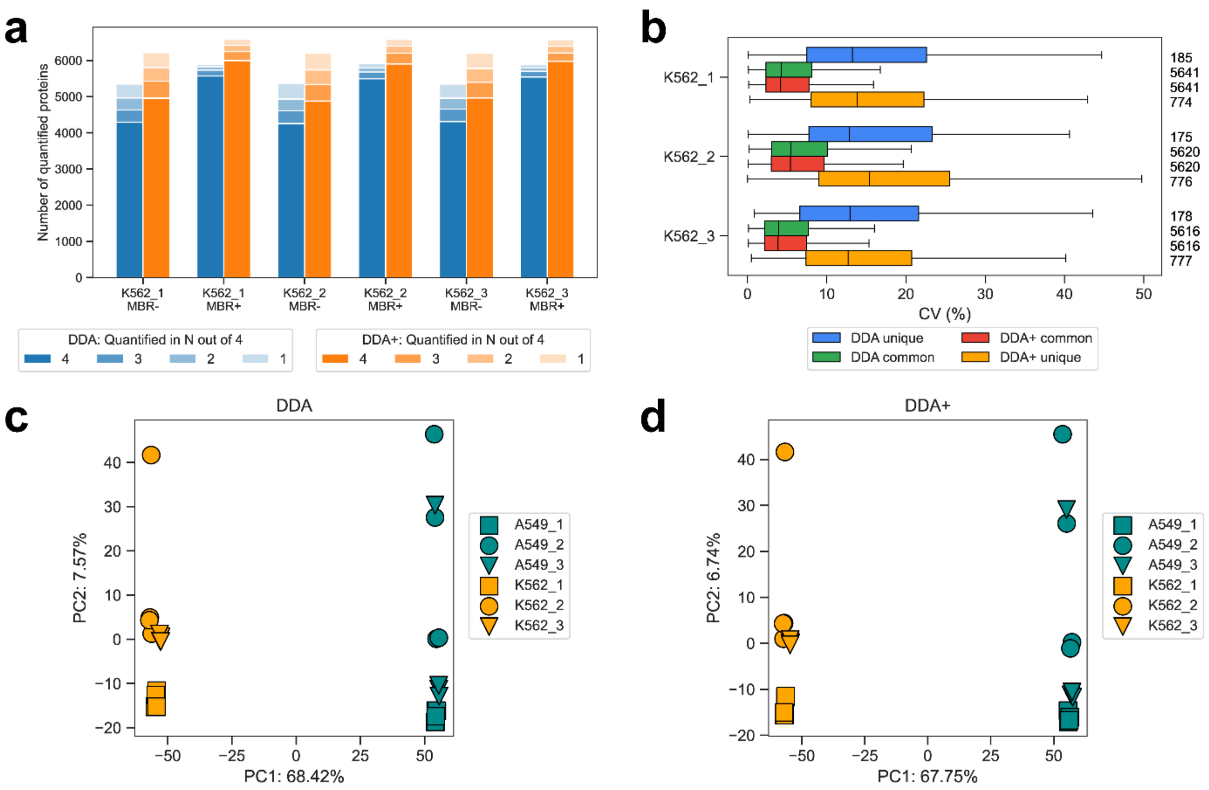

Supplementary Figure 3. Performance benchmarking using timsTOF ddaPASEF data. (a) Number of quantified proteins from the DDA and DDA+ workflows. The samples are from the K562 cell line. There are three biological replicates. Each biological replicate contains four technical replicates. “MBR+” and “MBR-” are with and without MBR, respectively. **(b)** Numbers and CVs of overlapped and non-overlapped proteins quantified from the DDA and DDA+ workflows using the K562 cell line. The blue box plots are from the unique proteins of the DDA mode, the green box plots are from the common proteins of the DDA mode, the red box plots are from the common proteins of the DDA+ mode, and the yellow box plots are from the unique proteins of the DDA+ mode. The common proteins are the overlapping proteins quantified in both DDA and DDA+ modes. The numbers on the right are the quantified proteins. For biological replicates K562_1, K562_2, and K562_3, there are 185, 175 and 178 unique proteins of the DDA mode; 774, 776, and 777 unique proteins of the DDA+ mode; and 5641, 5620, and 5616 common proteins, respectively. The box in each plot captures the interquartile range (IQR) with the bottom and top edges representing the first (Q1) and third quartiles (Q3), respectively. The median (Q2) is indicated by a horizontal line within the box. The whiskers extend to the minima and maxima within 1.5 times the IQR below Q1 or above Q3. **(c)** PCA plot of the quantitative results from two cell lines, A549 and K562, and their three experimental replicates. The result is from the DDA

workflow. **(d)** Similar to **(c)** but the result is from the DDA+ workflow. Source data are provided as a **Source Data** file.

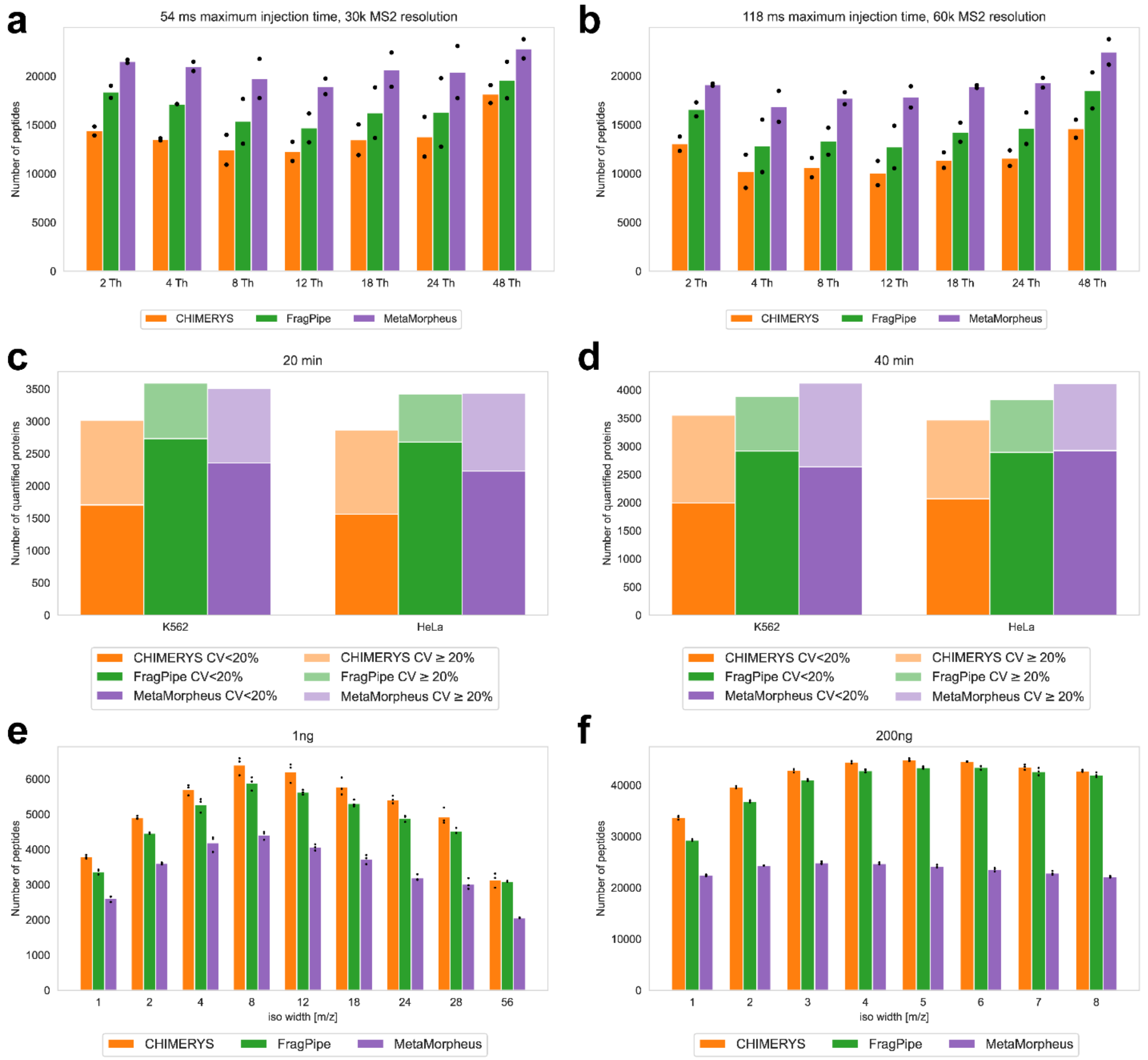

Supplementary Figure 4. Sensitivity assessment using three WWA datasets. (a) and **(b)** Numbers of peptides from the first WWA dataset. There are 14 samples with different isolation windows, maximum injection time, and MS2 resolutions. Each sample contain two technical replicates. MBR is enabled. The bar height represents the mean of the counts, and the black dot represents the peptide count for each replicate. **(c)** and **(d)** Numbers and CVs of quantified proteins from the second dataset. The samples are from K562 and HeLa cell lines, respectively. There are four samples with different combinations of cell line and gradient length. Each sample has eight technical replicates. MBR is enabled. **(e)** and **(f)** Numbers of identified peptides from the third WWA dataset. There are 17 samples with different isolation windows and sample amounts. Each sample has three technical replicates. The bar height represents the mean of the

counts, and the black dot represents the peptide count for each replicate. Source data are provided as a **Source Data** file.

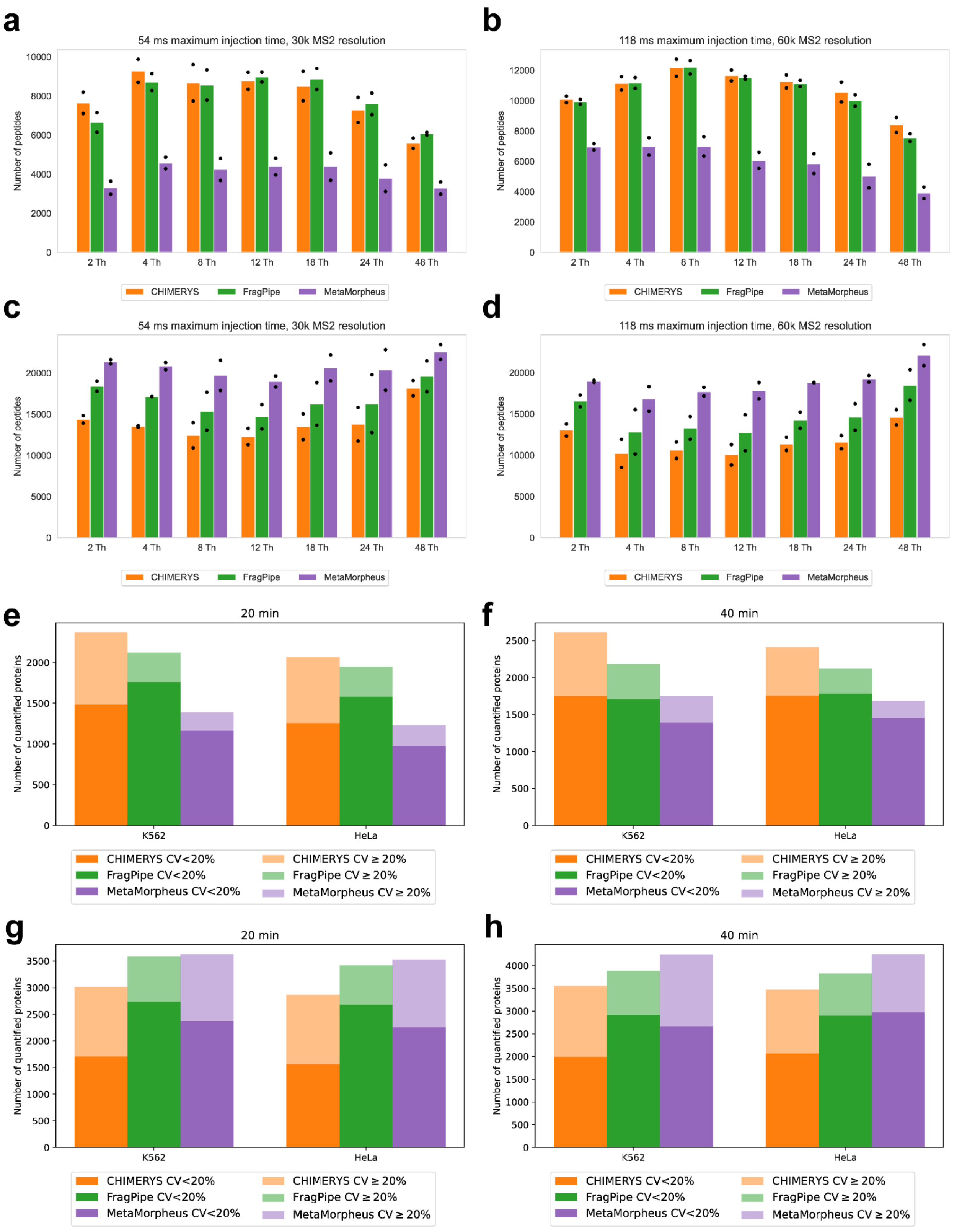

Supplementary Figure 5. Sensitivity assessment similar to Figure 4 and Supplementary Figure 3, but the latest version of MetaMorpheus (version 1.0.6) is used. (a) and **(b)** Numbers

of peptides from the first WWA dataset of Truong et al. MBR is disabled. **(c)** and **(d)** The same dataset as **(a)** and **(b)** but with MBR enabled. **(e)** and **(f)** Numbers and CVs of quantified proteins from the second dataset of Truong et al. MBR is disabled. **(g)** and **(h)** The same dataset as **(e)** and **(f)** but with MBR enabled. Source data are provided as a **Source Data** file.

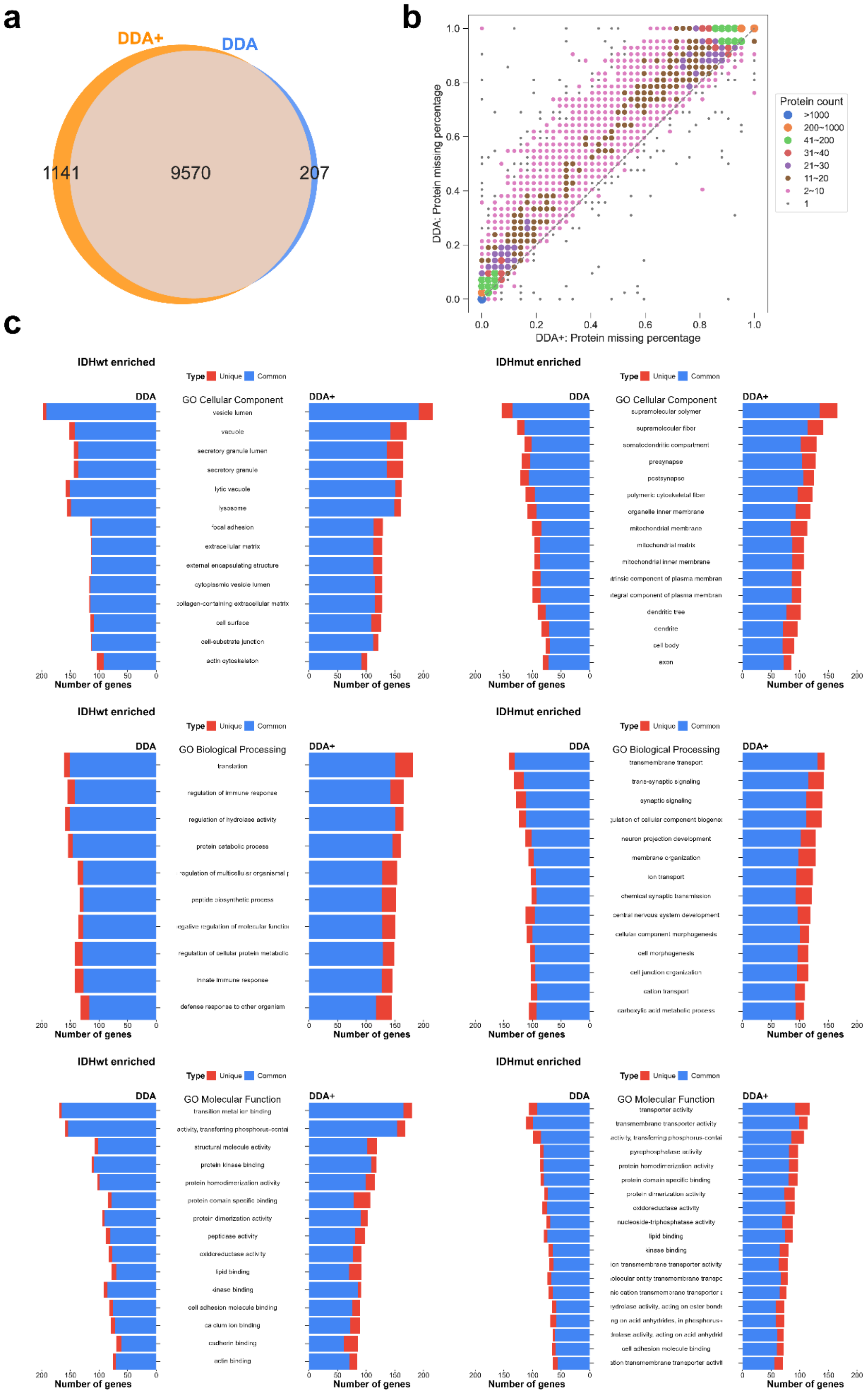

a
DDA+
DDA
1141
9570
207
b
DDA: Protein missing percentage
DDA+: Protein missing percentage
Protein count
>1000
200~1000
41~200
31~40
21~30
11~20
2~10
1
c
IDHwt enriched
IDHmut enriched
Type
Unique
Common
DDA
DDA+
Number of genes
GO Cellular Component
vesicle lumen
vacuole
secretory granule lumen
secretory granule
lytic vacuole
lysosome
focal adhesion
extracellular matrix
external encapsulating structure
cytoplasmic vesicle lumen
cell surface
cell-substrate junction
actin cytoskeleton
supramolecular polymer
supramolecular fiber
somatodendritic compartment
presynapse
postsynapse
polymeric cytoskeletal fiber
organelle inner membrane
mitochondrial membrane
mitochondrial matrix
mitochondrial inner membrane
dendritic tree
dendrite
cell body
axon
GO Biological Processing
translation
regulation of immune response
regulation of hydrolase activity
protein catabolic process
peptide biosynthetic process
regulation of cellular protein metabolic
innate immune response
defense response to other organism
transmembrane transport
trans-synaptic signaling
synaptic signaling
neuron projection development
membrane organization
ion transport
chemical synaptic transmission
central nervous system development
cellular component morphogenesis
cell morphogenesis
cell junction organization
cation transport
carboxylic acid metabolic process
GO Molecular Function
transition metal ion binding
structural molecule activity
protein kinase binding
protein homodimerization activity
protein domain specific binding
protein dimerization activity
peptidase activity
oxidoreductase activity
lipid binding
kinase binding
cell adhesion molecule binding
calcium ion binding
cadherin binding
actin binding
transporter activity
transmembrane transporter activity
pyrophosphatase activity
nucleoside-triphosphatase activity
ion transmembrane transporter activity

Supplementary Figure 6. Performance demonstration using a large-scale glioma dataset. **(a)** Venn diagram showing the number of quantified genes from the DDA and DDA+ workflows. **(b)** Scatter plot showing the percentage of protein-level missing values by comparing DDA and DDA+ workflows. **(c)** GO analysis using the results from the DDA and DDA+ workflows. Source data are provided as a **Source Data** file.
